# Sleep and Daytime Function in People with Spinal Cord Injury

**DOI:** 10.21203/rs.3.rs-4510393/v1

**Published:** 2024-06-28

**Authors:** Amira N. Badr, Salam Zeineddine, Anan Salloum, Nishtha Pandya, Michael N. Mitchell, Abdulghani Sankari, Isabel D. Muñoz, M. Safwan Badr, Jennifer L. Martin, Monica R. Kelly

**Affiliations:** 1. Department of Physical and Occupational Therapy, Boston Children’s Hospital, Boston, Massachusetts; 2. Department of Internal Medicine, Wayne State University, Detroit, MI; 3. John D. Dingell Veterans Affairs Medical Center, Detroit, MI; 4. Geriatric Research Education and Clinical Center, Veterans Affairs Greater Los Angeles Healthcare System, Los Angeles, CA; 5. Department of Medicine, University of California, Los Angeles, Los Angeles, CA

## Abstract

**Study Design:**

Cross-sectional cohort study.

**Objectives:**

To determine the role of sleep-disordered breathing (SDB), insomnia symptoms and sleep quality in the daytime function and quality of life of veterans with spinal cord injury (SCI).

**Setting:**

A Veterans Administration (VA) medical center in the Midwestern US.

**Methods:**

Thirty-eight male veterans with SCI (22 cervical, 16 thoracic; mean [SD] age = 62.9[9.5] years) completed baseline assessments within a larger clinical trial. Measures assessed sleep apnea severity (apnea-hypopnea index, AHI), insomnia symptoms (Insomnia Severity Index, ISI), self-reported sleep quality (Pittsburg Sleep Quality Index, PSQI), daytime sleepiness (Epworth Sleepiness Scale, ESS), fatigue (Flinders Fatigue Scale, FFS), depression (Patient Health Questionnaire-9 item, PHQ-9 excluding sleep item), functioning (Spinal Cord Independence Measure, SCIM), and quality of life (World Health Organization Quality of Life, WHOQOL-BREF). Bivariate correlations (alpha p<.05) were used to assess relationships between sleep (AHI, ISI, PSQI, ESS) and function (FFS, PHQ-9, SCIM, WHOQOL-BREF).

**Results:**

Mean AHI was 29.9(26.6), mean ISI was 9.38(6.2), mean PSQI was 9.0(4.6), and mean ESS was 7.0(5.2). There were no significant relationships between AHI and function measures. Significant relationships emerged between ISI and PHQ-9, some WHOQOL-BREF subscales, and SCIM as well as between PSQI and FFS, PHQ-9, and some WHOQOL-BREF subscales.

**Conclusions:**

Among Veterans with SCI, insomnia symptom severity and poor sleep quality were associated with worse functioning, whereas SDB severity was not. Insomnia and poor sleep quality represent modifiable contributors to poor daytime function. Research evaluating the impact of evidence-based insomnia treatments among individuals living with SCI is warranted.

## Introduction

Sleep disorders, such as sleep-disordered breathing (SDB) and insomnia disorder, are experienced by individuals living with spinal cord injury (SCI) at elevated rates as compared to the general population.(1) An estimated three-quarters of individuals with SCI meet the criteria for SDB,(2–4) and symptoms of insomnia are reported by the majority of individuals with spinal cord injuries.(5) Sleep problems may contribute to daytime symptoms such as fatigue, worse mental health symptoms, poor cognitive function, and impaired quality of life (6–8) in individuals with SCI who are already facing numerous challenges in their daily functioning.

SDB, the term for sleep disorders such as obstructive sleep apnea (OSA) that involve reduced or cessation of breathing during sleep, is often associated with myriad comorbidities and risk for significant health events (e.g., cardiovascular disease, stroke). However, insomnia may also be a cause for concern in the SCI population. Insomnia disorder, defined as chronic, habitual difficulty falling and/or staying asleep combined with daytime functional impairment,(9) can result in difficulty managing symptoms of other health conditions such as SCI and poor quality of life.

Our previous work demonstrated that the severity of insomnia symptoms is associated with elevated mental health symptoms of depression, anxiety, and posttraumatic stress disorder (PTSD) in individuals with spinal cord injuries and diseases (SCI/D).(10) Additionally, we found that both worse insomnia and worse sleep quality were linked to elevated daytime fatigue and reduced quality of life in individuals with multiple sclerosis (MS),(11) a spinal cord disease with functional challenges that may parallel those experienced by individuals with SCI.

While it is known that individuals with SCI have elevated rates of disturbed sleep, few studies have examined the relationships between sleep and functioning in individuals with SCI. Therefore, this investigation aimed to determine the relative contribution of SDB, insomnia symptoms, and self-reported sleep quality to daytime function and quality of life in veterans with SCI. We hypothesized that more severe SDB, worse insomnia symptoms, and worse sleep quality would be associated with worse daytime functioning.

## Methods

We analyzed data collected from a subset of participants enrolled in a larger clinical trial (NCT02830074) related to the treatment of SDB for individuals with either SCI or spinal cord disease (SCD).(12) Baseline assessments consisted of in-laboratory polysomnography (PSG) and questionnaires that assessed both daytime and nighttime functioning. Questionnaires were presented verbally via standardized scripts and visual response prompts to mitigate the impact of physical limitations experienced by many participants that could increase the burden of written or computer-based questionnaires. For the current analyses, we utilized data from the 38 veterans with SCI enrolled in the trial. The Institutional Review Board at Wayne State University in Detroit, MI, reviewed and approved the study. The VA Northeast Ohio Healthcare System also approved the study. A waiver of documentation of consent was obtained for screening. All participants gave written or witnessed verbal consent for those with limited upper extremity mobility. Study procedures took place at the John D. Dingell Veterans Administration Medical Center (JDDVAMC).

Inclusion criteria for the parent study were veterans with a spinal cord injury who were at least 3 months post-injury and were receiving care at one of the participating medical centers. Exclusion criteria were: mechanical ventilation, current use of positive airway pressure therapy (PAP) for SDB, clinical contraindication preventing PAP use, recent health event (e.g., recent stroke, recent surgery or hospitalization) that would likely impair sleep, less than 90 days sobriety from alcohol or substance abuse, self-reported inability to fully participate in study procedures (e.g., due to significant illness), or lack of capacity to self-consent (e.g., dementia).

### Study Measures:

#### Demographics:

Demographic data such as age, gender, race/ethnicity, marital status, employment status, living situation, and income were collected to describe the study sample.

#### Sleep Measures:

A single night of in-laboratory polysomnography (PSG) was performed to determine SDB diagnosis, severity via apnea hypopnea index (AHI: number of apneas and hypopneas per hour of sleep), and sleep efficiency (PSG-SE: percentage of time in bed spent asleep).(13) PSGs were scored according to AASM scoring criteria by trained technologists meeting AASM Interscorer reliability standards (13) and scoring was confirmed by a board-certified sleep medicine physician. PSG data were unavailable for 3 participants (n=35 for AHI and PSG-SE variables).

The Insomnia Severity Index (ISI) (14) was used to assess insomnia severity. The ISI is a subjective 7-item questionnaire that evaluates the quality of sleep, satisfaction with sleep, insomnia interference in daily functioning, severity of insomnia symptoms, distress caused by sleep problems, and if the insomnia is noticeable to others. The tool uses a Likert scale of 5 points. A score below 7 indicates no clinically significant insomnia, 8–14 as subthreshold insomnia, 15–21 as clinical insomnia (moderate severity), and 22–28 as clinical insomnia (severe).

The Pittsburgh Sleep Quality Index (PSQI) is a self-report questionnaire comprising 18 items assessing sleep habits and symptoms (i.e., sleep quality, latency, duration, efficiency, disturbances, daytime function, and medication use) over the past week. It is scored using both a 4-item Likert scale and open-ended questions converted to subscale scores. Higher scores are indicative of acute sleep disturbances. The PSQI has been shown to be a valid and reliable measure of good vs. poor sleepers (15). The three-subscale scoring system was used due to more favorable psychometric properties.(16)

#### Measures of Daytime Symptoms:

The Epworth Sleepiness Scale (ESS) (17) is a questionnaire that assesses the subjective likelihood of dozing off during 8 different daily activities such as reading, watching TV, or sitting as a passenger in a car on a scale from 0 (no chance of dozing) to 3 (high chance of dozing). The questionnaire is scored from 0–24 with a score over 10 indicating clinically significant sleepiness.

Flinder’s Fatigue Scale (FFS) (18) is a 7-item questionnaire that assesses the extent of daily fatigue in individuals with insomnia in frequency, severity, and impairment to daytime functioning. FFS scores range from 0 to 31. A score of 13–15 is borderline, 16–20 is moderate, and greater than 21 is severe fatigue.

The Patient Health Questionnaire-9 (PHQ-9) (19) is a commonly used questionnaire that assesses clinical depression symptoms in outpatient settings. It is scored from 0 to 27, and a score above 20 indicates clinically significant depression.

The Spinal Cord Independence Measure (SCIM) (20) is a 19-item questionnaire that assesses 3 domains of function for individuals with spinal cord injuries to determine their level of independence and measure functional progress. The first domain is self-care, which assesses feeding, bathing, dressing, and grooming (scored 0–20). The second domain is respiration and sphincter management, which consists of respiration and bladder and bowel management, and use of the toilet (scored 0–40). The third domain is mobility, which assesses tasks such as transfers from bed to wheelchair or wheelchair to toilet and mobility indoors and outdoors (scored 0–40). The total SCIM score ranges from 0–100, with higher scores indicating greater independence in functioning and assisting clinicians in developing a treatment plan, setting functional goals, and monitoring progress.

#### Quality of Life Measure:

The World Health Organization Quality of Life: Brief Version (WHOQOL-BREF) (21) is a self-report questionnaire assessing quality of life (QOL). The measure is an adapted, shorter version of the WHOQOL-100 questionnaire. It consists of 26 items that assess QOL in domains of physical health, psychological health, social relationships, and environment. Each question is scored on a 5-point Likert scale, with the mean score of each question determining the domain score and with higher scores indicative of higher QOL.

### Data Analysis:

Data were entered into REDCap (Research Electronic Data Capture) (22) and exported to Stata 17.0 (23) for analysis. Descriptive statistics were calculated for all study variables to contextualize the study sample. Relationships between sleep measures (AHI and SE from PSG, ISI, and PSQI) and daytime function (ESS, FFS, PHQ-9, WHOQOL, and SCIM) were assessed using bivariate correlations. An alpha level of ≤ .05 constituted statistical significance.

## Results

**Table 1** shows the characteristics of study participants. The mean age was 63 years, and 100% of the 38 participants were male.

**Table 2** summarizes the sleep, daytime symptoms, functioning, and quality of life measures. Of the 38 participants, sleep-disordered breathing (AHI ≥ 15 events/hour) was present in n=22 participants, and the sample mean AHI score was 30 events/hour. Insomnia, defined as ISI score ≥ 15, was present in n=8 (21.05%) participants. The sample mean ISI score was 9. Poor sleep quality, defined as PSQI score ≥ 5, was endorsed by n=28 (73.68%) participants. The sample mean PSQI score was 9.

**Table 3** summarizes the correlations between sleep, daytime symptoms, functioning, and quality of life measures. There were no significant relationships between AHI and any daytime symptoms, SCI-related functioning, or quality of life outcomes. Worse objective sleep quality (PSG-SE) only correlated with poorer SCI-related functioning (SCIM). Significant relationships were observed between greater insomnia symptoms (ISI) and worse depression symptoms (PHQ-9), quality of life (both the Physical Health and Environment subscales of the WHOQOL-BREF), and functioning (SCIM). Lower self-reported sleep quality (PSQI) was associated with worse fatigue (FFS), depression symptoms (PHQ-9), and quality of life (three subscales of the WHOQOL-BREF: Physical Health, Psychological Health, and Environment subscales). Higher daytime sleepiness (ESS) correlated with worse fatigue (FFS), depression symptoms (PHQ-9), quality of life (all four subscales of the WHOQOL-BREF: Physical Health, Psychological Health Social Relationships, and Environment), and SCI-related functioning (SCIM).

## Discussion

### Summary of Findings

These analyses aimed to expand the existing literature on the interplay between sleep (specifically, SDB, insomnia symptoms, and perceived sleep quality), functioning, and quality of life in Veterans with SCI. Our study demonstrated that veterans living with SCI have high rates of sleep disturbance (insomnia symptoms and self-reported sleep quality), which was significantly associated with worse daytime function and quality of life. However, our hypothesis that sleep apnea severity (AHI) would also be associated with daytime symptoms, quality of life, and functioning outcomes was not confirmed. Our data suggest poor sleep and insomnia symptoms, regardless of SDB status, are critical determinants of daytime impairments.

### Determinants of Sleep Quality in Veterans Living with SCI

We found that self-reported measures of sleep quality, specifically PSQI and ISI, were related to depression, quality of life, and SCIM. Depression is common in individuals living with SCI,(24) and they often experience lower life satisfaction.(25) The determinants of depression in this population are not clear; however, poor sleep quality can result in less engagement in daily activities and rehabilitation, decreasing quality of life and overall independence,(26) which may contribute to depression symptoms.

Rehabilitation literature corroborates our findings that poor sleep can lead to high levels of fatigue that diminishes engagement in daily meaningful activities for individuals with SCI.(27) Continued poor sleep can result in limited engagement in rehabilitation activities, resulting in decreased daily functioning of those with SCI.(28) Sleep disturbance and poor sleep patterns can increase the energy and time required for daily SCI care and participation in functional tasks, thereby limiting energy for engagement in other meaningful activities. Rehabilitation clinicians may focus on other risk factors for poor outcomes in those with SCI, such as bowel/bladder, pressure sores, and decreased function. Unfortunately, sleep may be overlooked in the rehabilitation setting despite the knowledge that poor sleep is linked to negative outcomes in individuals with SCI.(29) Therefore, poor sleep may be a modifiable risk factor to improve not only general function but also mood and mental health conditions such as depression in veterans with SCI.

### Limitations

Several methodological considerations may affect the interpretation of our findings. First, the study sample size was small, which precluded multivariable modeling and increased the risk of type 2 errors. As such, we avoided interpreting any one statistical test but focused on the pattern of results (i.e., that insomnia but not SDB severity was associated with functional impairments). Second, the diagnostic sleep studies were conducted in a sleep laboratory and do not represent typical sleep in the home environment. However, in-laboratory polysomnography remains the gold standard for assessing sleep architecture and diagnosing SDB among individuals with musculoskeletal abnormalities, such as those with SCI. Third, the generalizability of the study is limited to male veterans with SCI, which may not be representative of women or non-veterans with SCI. Finally, circadian rhythm disorder is common in those with cervical spinal cord injury owing to a lack of melatonin secretion in this population, and our study did not include a formal assessment of circadian tendencies or disorders.(30)

### Implications of Findings

Sleep quality and disorders should be prioritized in the overall comprehensive care of individuals living with SCI. Investigating causes of poor sleep quality in individuals living with SCI will include causes of sleep fragmentation (including SDB), poor sleep quality, and insomnia disorder, with the aim of identifying modifiable risk factors. Cognitive behavioral therapy for insomnia (CBT-I) is the first-line treatment for chronic insomnia disorder; however, CBT-I has not been systematically evaluated among patients with SCI, and adaptations to standard treatment components may be necessary. For example, stimulus control instructs a patient to move out of the bedroom if unable to fall asleep; however, this can be a significant challenge for those living with SCI, and adaptations that do not increase the burden on family care providers are needed.

## Conclusion

Sleep disturbance, specifically insomnia symptom severity and poor sleep quality, were associated with worse daytime functioning in Veterans with SCI. However, SDB was not linked to any functioning measures. Improvement of insomnia and sleep quality may benefit daytime function; therefore, assessing evidence-based treatments for insomnia, such as cognitive-behavioral therapy, is necessary in individuals with SCI.

## Data Availability

Data are available upon request from the corresponding author.

## References

[1] Fogelberg DJ, Hughes AJ, Vitiello MV, Hoffman JM, Amtmann D. Comparison of Sleep Problems in Individuals with Spinal Cord Injury and Multiple Sclerosis. J Clin Sleep Med. 2016;12(5):695–701. 26857058 10.5664/jcsm.5798 PMC4865556

[2] Sankari A, Vaughan S, Bascom A, Martin JL, Badr MS. Sleep-Disordered Breathing and Spinal Cord Injury: A State-of-the-Art Review. Chest. 2019;155(2):438–45. 30321507 10.1016/j.chest.2018.10.002 PMC6688981

[3] Sankari A, Martin JL, Badr MS. Sleep Disordered Breathing and Spinal Cord Injury: Challenges and Opportunities. Curr Sleep Med Rep. 2017;3(4):272–8. 29177130 10.1007/s40675-017-0093-0 PMC5697765

[4] Giannoccaro MP, Moghadam KK, Pizza F, Boriani S, Maraldi NM, Avoni P, Sleep disorders in patients with spinal cord injury. Sleep medicine reviews. 2013;17(6):399–409. 23618534 10.1016/j.smrv.2012.12.005

[5] Shafazand S, Anderson KD, Nash MS. Sleep Complaints and Sleep Quality in Spinal Cord Injury: A Web-Based Survey. J Clin Sleep Med. 2019;15(5):719–24. 31053202 10.5664/jcsm.7760 PMC6510683

[6] Knutson KL, Van Cauter E. Associations between sleep loss and increased risk of obesity and diabetes. Ann N Y Acad Sci. 2008;1129:287–304. 18591489 10.1196/annals.1417.033 PMC4394987

[7] Kyle SD, Morgan K, Espie CA. Insomnia and health-related quality of life. Sleep medicine reviews. 2010;14(1):69–82. 19962922 10.1016/j.smrv.2009.07.004

[8] LeBlanc M, Merette C, Savard J, Ivers H, Baillargeon L, Morin CM. Incidence and risk factors of insomnia in a population-based sample. Sleep. 2009;32(8):1027–37. 19725254 10.1093/sleep/32.8.1027 PMC2717193

[9] Diagnostic and statistical manual of mental disorders : DSM-5. American Psychiatric A, American Psychiatric Associations DSMTF, editors. Arlington, VA: American Psychiatric Association; 2013.

[10] Kelly MR, Zeineddine S, Mitchell MN, Sankari A, Pandya N, Carroll S, Insomnia severity predicts depression, anxiety, and posttraumatic stress disorder in veterans with spinal cord injury or disease: a cross-sectional observational study. J Clin Sleep Med. 2023;19(4):695–701. 36661092 10.5664/jcsm.10410 PMC10071376

[11] Aljundi NA, Kelly M, Zeineddine S, Salloum A, Pandya N, Shamim-Uzzaman QA, Sleep disorders, daytime symptoms, and quality of life in veterans with multiple sclerosis: preliminary findings. Sleep Adv. 2022;3(1):zpac012. 10.1093/sleepadvances/zpac012 PMC10104398 37193412

[12] Badr MS, Martin JL, Sankari A, Zeineddine S, Salloum A, Henzel MK, Intensive support does not improve PAP use in Spinal Cord injury/disease: A randomized clinical trial. Sleep. 2024. 10.1093/sleep/zsae044 PMC11494382 38422375

[13] Berry RB, Brooks R, Gamaldo C, Harding SM, Lloyd RM, Quan SF, AASM Scoring Manual Updates for 2017 (Version 2.4). J Clin Sleep Med. 2017;13(5):665–6. 28416048 10.5664/jcsm.6576 PMC5406946

[14] Bastien CH, Vallieres A, Morin CM. Validation of the Insomnia Severity Index as an outcome measure for insomnia research. Sleep Medicine. 2001;2(4):297–307. 11438246 10.1016/s1389-9457(00)00065-4

[15] Backhaus J, Junghanns K, Broocks A, Riemann D, Hohagen F. Test–retest reliability and validity of the Pittsburgh Sleep Quality Index in primary insomnia. J Psychosom Res. 2002;53(3):737–40. 12217446 10.1016/s0022-3999(02)00330-6

[16] Cole JC, Motivala SJ, Buysse DJ, Oxman MN, Levin MJ, Irwin MR. Validation of a 3-factor scoring model for the Pittsburgh sleep quality index in older adults. Sleep. 2006;29(1):112–6. 16453989 10.1093/sleep/29.1.112

[17] Johns MW. A New Method for Measuring Daytime Sleepiness: The Epworth Sleepiness Scale. Sleep. 1991;14(6):540–5. 1798888 10.1093/sleep/14.6.540

[18] Gradisar M, Lack L, Richards H, Harris J, Gallasch J, Boundy M, The Flinders Fatigue Scale: preliminary psychometric properties and clinical sensitivity of a new scale for measuring daytime fatigue associated with insomnia. J Clin Sleep Med. 2007;3(7):722–8. 18198807 PMC2556916

[19] Levis B, Benedetti A, Thombs BD, Collaboration DESD. Accuracy of Patient Health Questionnaire-9 (PHQ-9) for screening to detect major depression: individual participant data meta-analysis. BMJ. 2019;365:l1476–l. 30967483 10.1136/bmj.l1476 PMC6454318

[20] Catz A, Itzkovich M, Agranov E, Ring H, Tamir A. SCIM--spinal cord independence measure: a new disability scale for patients with spinal cord lesions. Spinal Cord. 1997;35(12):850–6. 9429264 10.1038/sj.sc.3100504

[21] Development of the World Health Organization WHOQOL-BREF quality of life assessment. The WHOQOL Group. Psychol Med. 1998;28(3):551–8. 9626712 10.1017/s0033291798006667

[22] Harris PA, Taylor R, Thielke R, Payne J, Gonzalez N, Conde JG. Research electronic data capture (REDCap)--a metadata-driven methodology and workflow process for providing translational research informatics support. Journal of biomedical informatics. 2009;42(2):377–81. 18929686 10.1016/j.jbi.2008.08.010 PMC2700030

[23] StataCorp. Stata Statistical Software. 17 ed. College Station, TX: StataCorp LLC.; 2021.

[24] Hoffman JM, Bombardier CH, Graves DE, Kalpakjian CZ, Krause JS. A longitudinal study of depression from 1 to 5 years after spinal cord injury. Arch Phys Med Rehabil. 2011;92(3):411–8. 21353823 10.1016/j.apmr.2010.10.036

[25] Post MW, van Leeuwen CM. Psychosocial issues in spinal cord injury: a review. Spinal Cord. 2012;50(5):382–9. 22270190 10.1038/sc.2011.182

[26] Sankari A, Badr MS, Martin JL, Ayas NT, Berlowitz DJ. Impact Of Spinal Cord Injury On Sleep: Current Perspectives. Nat Sci Sleep. 2019;11(11 ):219–29. 31686935 10.2147/NSS.S197375 PMC6800545

[27] Widerstrom-Noga EG, Felipe-Cuervo E, Yezierski RP. Chronic pain after spinal injury: interference with sleep and daily activities. Arch Phys Med Rehabil. 2001;82(11):1571–7. 11689978 10.1053/apmr.2001.26068

[28] Tester NJ, Foss JJ. Sleep as an Occupational Need. Am J Occup Ther. 2018;72(1):7201347010p1-p4. 10.5014/ajot.2018.020651 29280728

[29] Fogelberg DJ, Leland NE, Blanchard J, Rich TJ, Clark FA. Qualitative Experience of Sleep in Individuals With Spinal Cord Injury. OTJR (Thorofare N J). 2017;37(2):89–97. 28196449 10.1177/1539449217691978 PMC5447661

[30] Whelan A, Halpine M, Christie SD, McVeigh SA. Systematic review of melatonin levels in individuals with complete cervical spinal cord injury. J Spinal Cord Med. 2020;43(5):565–78. 30132738 10.1080/10790268.2018.1505312 PMC7534275

